# 47. Transitioning to Batch Dosing of High-Cost Antimicrobials in the Inpatient Setting

**DOI:** 10.1093/ofid/ofab466.249

**Published:** 2021-12-04

**Authors:** Matthew Lokant, Shu Xian Lee, Connor Nevin, John D Lindsay, Colby Acri, Miles Graves, Katherine L Bergman, John Guilfoose, Catessa Howard

**Affiliations:** 1 West Virginia University, Morgantown, West Virginia; 2 WVU Medicine, Morgantown, West Virginia

## Abstract

**Background:**

Antimicrobial stewardship (AMS) committees ensure appropriate antimicrobial utilization. One stewardship intervention is to evaluate the delivery model of high-cost antimicrobials to better utilize resources and mitigate expenses. We analyzed the total medication waste and costs of high-cost antimicrobials, specifically daptomycin, ertapenem, amphotericin, and micafungin, at our institution and propose innovative cost-savings changes at a systems level.

**Methods:**

This retrospective study consisted of 263 patients. All patients were at least 18 years old who was admitted to our academic institution from January 2020 to April 2021 and received daptomycin, ertapenem, amphotericin, or micafungin. Demographics, daily medication dosage, total doses received, the date and time of the start of the medication, last administered dose, and discontinued order were recorded.

**Results:**

The daptomycin cohort consisted of 143 patients with 46.2% females and average age of 56.3 years. In this group, 145.3 vials were wasted which equated to a loss of &22,630. The ertapenem group had 53 patients with 62.3% females and a mean age of 62.3 years. There were 24 vials wasted with a calculated loss of &1080. The amphotericin cohort had 32 patients with an average age of 52.2 years and 43.8% females. There were 189 vials wasted with a loss of &46,116. The micafungin group had 35 patients with 42.9% females and average age of 60.4 years. This group had 12 vials wasted with a loss of &2052.

**Conclusion:**

Each antimicrobial has a specific formulation protocol. Daptomycin and ertapenem formulation occurs in the early morning. Amphotericin formulation occurs 2 hours prior to medication use. Micafungin formulation occurs at the time the order label prints. These medications were more often administered in the late morning to early afternoon timeframe. The order to discontinue the medications also occurred at the same interval. One reason could be due to decisions made on morning rounds from primary teams and specialty input. These orders would then be placed after rounds. A cost-saving method would be to batch and change the formulation time for all antimicrobials to later in the afternoon, which would not only prevent waste, but also allow the AMS team to effectively audit appropriate antimicrobial use.

**Disclosures:**

**All Authors**: No reported disclosures

